# Title IgA Nephropathy and Oral Bacterial Species Related to Dental Caries and Periodontitis

**DOI:** 10.3390/ijms23020725

**Published:** 2022-01-10

**Authors:** Yasuyuki Nagasawa, Taro Misaki, Seigo Ito, Shuhei Naka, Kaoruko Wato, Ryota Nomura, Michiyo Matsumoto-Nakano, Kazuhiko Nakano

**Affiliations:** 1 Department of General Internal Medicine, Hyogo College of Medicine, Nishinomiya 663-8501, Hyogo, Japan; 2 Division of Nephrology, Seirei Hamamatsu General Hospital, Hamamatsu 430-8558, Shizuoka, Japan; misakitar@gmail.com; 3 Department of Nursing, Faculty of Nursing, Seirei Christopher University, Hamamatsu 433-8558, Shizuoka, Japan; 4 Department of Internal Medicine, Japan Self-Defense Gifu Hospital, Kakamigahara 502-0817, Gifu, Japan; seigoemon@yahoo.co.jp; 5 Department of Pediatric Dentistry, Okayama University Graduate School of Medicine, Dentistry and Pharmaceutical Sciences, Okayama 700-8525, Okayama, Japan; nshuhei@okayama-u.ac.jp (S.N.); mnakano@cc.okayama-u.ac.jp (M.M.-N.); 6 Department of Pediatric Dentistry, Division of Oral Infection and Disease Control, Osaka University Graduate School of Dentistry, Suita 565-0871, Osaka, Japan; wato@dent.osaka-u.ac.jp (K.W.); rnomura@dent.osaka-u.ac.jp (R.N.); nakano@dent.osaka-u.ac.jp (K.N.)

**Keywords:** IgA nephropathy, periodontal bacteria, dental caries, periodontitis, infection, mouse, tonsil, oral bacteria, *Porphyromonas gingivalis*, *cnm*, *Streptococcus mutans*

## Abstract

A relationship between IgA nephropathy (IgAN) and bacterial infection has been suspected. As IgAN is a chronic disease, bacteria that could cause chronic infection in oral areas might be pathogenetic bacteria candidates. Oral bacterial species related to dental caries and periodontitis should be candidates because these bacteria are well known to be pathogenic in chronic dental disease. Recently, several reports have indicated that collagen-binding protein (*cnm*)-(+) *Streptococcs mutans* is relate to the incidence of IgAN and the progression of IgAN. Among periodontal bacteria, *Treponema denticola*, *Porphyromonas gingivalis* and *Campylobacte rectus* were found to be related to the incidence of IgAN. These bacteria can cause IgAN-like histological findings in animal models. While the connection between oral bacterial infection, such as infection with *S. mutans* and periodontal bacteria, and the incidence of IgAN remains unclear, these bacterial infections might cause aberrantly glycosylated IgA1 in nasopharynx-associated lymphoid tissue, which has been reported to cause IgA deposition in mesangial areas in glomeruli, probably through the alteration of microRNAs related to the expression of glycosylation enzymes. The roles of other factors related to the incidence and progression of IgA, such as genes and cigarette smoking, can also be explained from the perspective of the relationship between these factors and oral bacteria. This review summarizes the relationship between IgAN and oral bacteria, such as *cnm*-(+) *S. mutans* and periodontal bacteria.

## 1. Introduction

**Figure 1:**
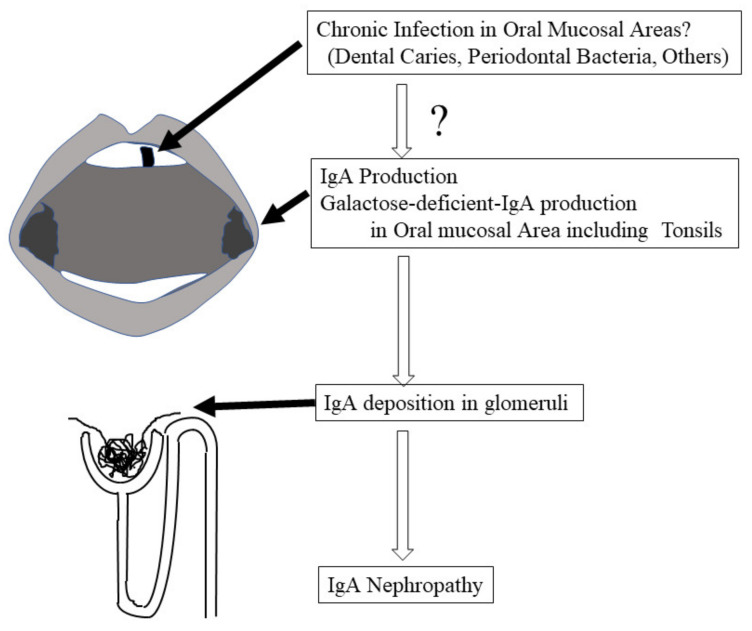
**Cascade from oral infections to IgAN.** IgA nephropathy was defined by the deposition of IgA in the mesangial area of the glomeruli. This IgA might be produced in oral area mucosa lymphoid tissues, including the tonsils, which are called NALT. The most suspicious stimulator of IgA production by NALT might be chronic oral area infections, such as bacteria related to dental caries and periodontal bacteria.

IgA nephropathy (IgAN) is the most common primary glomerulonephritis [1,2,3,4], and its pathogenesis remains unclear [5]. Similar to other chronic kidney diseases, many atherosclerotic factors, such as hypertension, uric acid [6], cigarette smoking [7], and kidney function itself, promote the progression of IgAN. IgAN is defined as the deposition of IgA in mesangial areas in glomeruli, and the subclass of IgA in this deposition is IgA1, which may be produced in mucosal areas in the upper respiratory tract, including the tonsils or mucosal tissues in oral areas. Moreover, tonsillectomy has been reported to ameliorate disease progression [8,9,10]. These findings strongly indicate that the kidney deposition of IgA in IgAN patients might come from oral areas, including the tonsils (see Figure 1). IgA in the mucosal areas is produced to eliminate exogeneous antigens. Although IgAN patients sometimes manifest with macrohematuria just after acute tonsilitis, the disease progression itself to renal failure must require more than 20–30 years. Therefore, the stimulation of IgA production may also continue for more than 20–30 years, which increases or decreases in some periods. The strongest candidate as this stimulator is chronic infection in the oral areas, such as bacterial species related to dental caries and periodontitis (see Figure 1). These bacterial species have been reported to have a strong relationship with many systemic diseases [11,12,13,14,15,16], and these bacteria participate in the pathogenesis of dental diseases, such as dental caries and periodontal disease. Recently, several reports indicated that these bacterial species may be involved in the pathogenesis of IgAN as a stimulator of IgA production (see Figure 1). In this review, we summarize and discuss the possibility that these bacterial species are part of the pathogenesis of IgAN and the possible mechanisms by which these oral bacteria cause IgAN.

## 2. Historical Findings

In terms of the relationship between oral bacterial infection and primary glomerulonephritis, the most established kidney disease is acute glomerulonephritis (AGN) [17,18]. In one study, approximately two weeks after patients had group A beta-*Streptococcus* tonsillar infection as a preceding infection, patients presented with macrohematuria, proteinuria, hypertension, edema, and typical glomerular lesions, such as endocapillary proliferation and subepithelial hump, which can be proven by kidney biopsy. Immunohistochemical staining revealed IgG and C3 deposition along with glomerular capillaries. Antibodies to *Streptococcus*, such as anti-streptolysin O antibody (ASO) and anti-streptokinase antibody (ASK), can prove *Streptococcus* tonsillar infection, resulting in the diagnosis of AGN [17]. Treatment of *Streptococcus* infection with antibiotics can ameliorate kidney lesions at the same time. After patients with AGN have recovered from *Streptococcus* tonsillar infection, kidney phenomena will also usually disappear without specific treatment for proteinuria or hematuria, although in some cases, recovery from AGN is delayed. To investigate the pathogenesis of AGN, a cultural method to diagnose *Streptococcus* infection using tonsillar swabs may work because *Streptococcus* belong to the aerobic bacteria. However, there are many anaerobic oral bacteria that cannot be detected by the culture method.

IgAN patients sometimes manifest with macrohematuria just after they develop tonsillar infection. This phenomenon indicates that there is some association between IgAN and oral area infection, especially tonsillitis. Moreover, the subclass of IgA deposited in the mesangial area of kidney biopsy specimens is well known to be IgA1, which is usually induced in the upper respiratory tract, including oral mucosal areas, rather than in the gastrointestinal tract. IgA1 is produced to attack bacterial infection in the mucosal areas. These facts also support that bacterial infection in the mucosal areas might be a strong candidate for the pathogenesis of IgAN. Suzuki-S et al. reported that the antigen of *Haemophilus parainfluenzae* can be detected in kidney biopsy specimens of IgAN patients, while the antigen could not be detected in patients with other primary glomerulonephritis [19]. This group confirmed the production of IgA against *Haemophilus parainfluenzae* in tonsillar lymphocytes [20]. Administration of the antigen of *Haemophilus parainfluenzae* to mice can induce mesangial proliferation and glomerular IgA deposition in mouse kidneys 30–40 weeks after the start of treatment [21]. However, IgAN can persist for more than a decade, while tonsilitis induced by Haemophilus parainfluenzae recovers within one month. Chronic *Haemophilus parainfluenzae* infection in the tonsils has not been reported. Moreover, there are many aerobic and anaerobic bacterial infections that occur in the tonsils and in other oral areas; therefore, *Haemophilus parainfluenzae* remains a candidate pathogen of IgAN.

## 3. Nasopharynx-Associated Lymphoid Tissue (NALT) and Gut-Associated Lymphoid Tissue (GALT)

Since IgAN is exacerbated after mucosal infections affecting the upper respiratory tract and gastrointestinal tract, the involvement of mucosal immunity in its pathogenesis has been postulated [22,23]. This hypothesis is supported by the fact that IgA is an immunoglobulin that predominantly functions in the mucosa, that glomerular IgA is a mucosal-type multimeric IgA1 containing a secretory component, and that patients with IgAN have increased levels of multimeric IgA1 in the circulating blood [24,25,26,27,28]. The mucosal-associated lymphoid organ (MALT) is responsible for synthesizing most of the total IgA in the whole body, and it has been reported that the amount of IgA produced by the mucosal surface in a day is greater than the total amount of all other types of antibodies [29,30]. However, it is unclear which mucosal tissues of MALT are primarily involved in the pathogenesis of IgAN. The exacerbation of IgAN after upper respiratory tract infection suggests that nasopharynx-associated lymphoid tissue (NALT) is involved in IgAN. On the other hand, since the gut-associated lymphoid tissue (GALT) has a large mucosal area and is the main site of mucosal IgA production, which is involved in the pathogenesis of IgAN, and is controversial. Moreover, the exacerbation of IgAN after mucosal infection suggests the involvement of exogenous antigens; nevertheless, it is also unclear how infection plays a role in IgAN exacerbation. Serum IgA is produced mainly in the bone marrow, and serum IgA produced from the intestinal mucosa is thought to be low. On the other hand, a large amount of IgA is produced and secreted by the mucosa in the GALT, which contributes to intestinal immunity. Unlike mucosal IgA, the physiological role of serum IgA remains unclear. There are two subclasses of human IgA, namely, IgA1 and IgA2, and approximately 80% of serum IgA is IgA1 [31]. IgA1 shows abnormal glycosylation of O-glycans in its hinge region and is mainly present as the polymeric form in patients with IgAN [32,33,34,35,36]. The synthesis and binding of autoantibodies against galactose-deficient IgA1 (Gd-IgA1) are required for the formation of immune complexes (ICs), which accumulate in the mesangium, activate mesangial cell proliferation and matrix expansion, and cause glomerular injury [37]. Therefore, Gd-IgA1 may play a central role as a first hit in the pathogenesis of IgAN. The distribution of IgA1-positive and IgA2-positive plasma cells in the mucosa shows that IgA2-positive plasma cells are more prevalent in the intestinal mucosa (30–65%) than in the peripheral lymph nodes and airway mucosa (7–25%), with IgA2-positive plasma cells predominating, particularly in the ileum and colon [31,38,39,40]. It is known that the mechanisms of organogenesis of the Peyer’s patch (PP), the central organ of the GALT, and the NALT are very different. In the PP, CD3-negative CD4-positive CD45-positive inducer cells increase during the fetal period and decrease during the first 3 weeks of life, whereas in NALT, these cells increase with postnatal stimulation with foreign antigens, peaking at 3 weeks of life [41,42]. Furthermore, the molecular mechanisms involved in the formation of these organs are different [42]. These facts suggest that the basic roles and needs of both in mucosal immunity are different. In addition, microbial infections of mucous membranes may affect various autoimmune responses, and several reports have shown that the composition of the bacterial flora, especially in the intestinal tract, tonsils, saliva, and gingival margin, differs between patients with IgAN and controls [43,44,45,46,47,48]. The involvement of mucosal exposure to exogenous antigens, such as fungal, bacterial, and viral antigens, in the pathogenesis of IgAN makes it even more difficult to answer the question of which MALT sites are most involved in the pathogenesis of IgAN.

**Figure 2:**
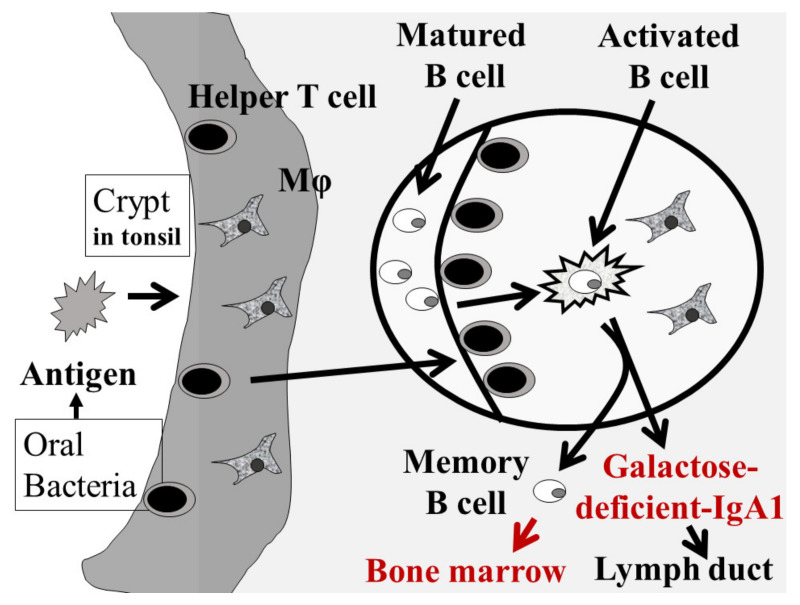
**IgA1 production in the tonsils.** In the crypt of the tonsils, macrophages/dendritic cells phagocytose antigens, such as oral bacteria, and present antigens to helper T cells. Activated helper T cells stimulate and activate mature B cells in the tonsillar germinal center to produce Gd-IgA1. The produced Gd-IgA1 flows into the systemic circulation via the lymphatic system. At that time, the activated B cells migrate into the body circulation and settle in the bone marrow.

Human NALT is an immune-guided tissue consisting mainly of Waldeyer’s pharyngeal ring, located at the entrance to the upper respiratory tract and gastrointestinal tract, and serves as the first line of defense against foreign antigens. Waldeyer’s pharyngeal ring is composed of the pharyngeal tonsils (adenoids), Eustachian tonsils, palatine tonsils, and lingual tonsils. Anatomical features, such as the location and morphology of NALT, differ between humans and mice [42]. Although mouse NALT does not have adenoids or palatine tonsils, it has developed mucosa-induced lymphoid tissue in the floor of the nasal cavity. More than 95% of IgA-positive plasma cells in human NALT are IgA1-producing, which is very different from GALT [49]. IgA produced in NALT is dimerized by the J chain and secreted to the mucosal surface via the polymeric immunoglobulin receptor (pIgR), which is responsible for transporting IgA and IgM produced in the mucosa to the mucosal surface, and this is also the case for GALT [31,40,49]. Since intranasal vaccine antigen sensitization of pIgR knockout mice results in an increase in antigen-specific IgA in the blood, pIgR dysfunction in NALT is thought to result in an increase in mucosal-type IgA in the blood [41]. Furthermore, it is important to note that IgAN is frequently associated with chronic tonsillitis and that the J-chain expression rate of IgA-positive plasma cells is decreased in both the germinal center (GC) and extrafollicular regions in human chronic tonsillitis [49,50]. Among Waldeyer’s pharyngeal rings, the palatine tonsils, in particular, play an important role in the antigen-specific immune induction of B cells within the developing lymphoid follicles, with their deeply branched crypts increasing the surface area and the actively sampling of foreign antigens (see Figure 2). In the dark zone, located in the GC of the palatine tonsils, automatic hypermutation and clonal expansion of B cells occur. On the other hand, in the light zone, antigen selection and cell proliferation occur, and effector B cells and memory B cells are induced, which then prepare for antigen-specific antibody production (see Figure 2). After homing to mucosal tissues, the induced effector B cells differentiate into plasma cells and exert mucosal defense functions through the production of various types of immunoglobulins in the local mucosa. Since the deep part of the crypt is an anaerobic environment, periodontal bacteria, which are anaerobic, can take hold. In addition, oral bacteria and their components act as antigens that are presented to immune cells in this environment. Furthermore, IgAN patients who underwent tonsillectomy tended to have normalized glycosylation abnormalities in the IgA hinge region, suggesting that NALT is important for Gd-IgA1 production [51]. It is known that B cells induced by NALT and GALT differ greatly in their homing tendencies [52,53]. B cells immunologically induced in GALT tend to home only to GALT, whereas B cells immunologically induced in NALT not only home to NALT and bronchus-associated lymphoid tissue (BALT) but also home to mucosa and lymphoid tissue throughout the body and tend to be widely involved in immune responses [42,49]. The dichotomy of B-cell homing is defined by the combination of integrin and chemokine receptor patterns expressed by effector B cells induced in NALT and GALT, respectively, and the patterns of adhesion molecules expressed in mucosal tissues [49,53]. In other words, immunosensitization targeting NALT efficiently induces antigen-specific immunity not only in the upper respiratory tract but also in the whole body, while immunosensitization targeting PP, representing GALT, is thought to work mainly for immune defense of the gastrointestinal mucosa [42,49].

## 4. NALT and IgA Nephropathy

Currently, Gd-IgA1 immunocomplexes generated by galactose-deficient IgA (Gd-IgA1) and endogenous anti-Gd-IgA1 antibodies are considered to be the effector molecules that cause IgAN [37]. Although it is still inconclusive which tissues among NALT and GALT are the major mucosal sites involved in the production of nephropathogenic IgA, recent clinical studies have shown the efficacy of tonsillectomy in patients with IgAN [10]. Sato et al., reported that in patients with IgAN, tonsillectomy alone reduced serum IgA levels by an average of approximately 9% [54]. This finding suggests that the main source of IgA in patients with IgAN is the tonsils, which are part of the NALT. Furthermore, when Gd-IgA1 levels were measured before and after tonsillectomy, it was confirmed that hematuria was significantly improved early after tonsillectomy in patients whose Gd-IgA1 levels decreased after tonsillectomy alone [55]. These results suggest that the palatine tonsils may be the major site of Gd-IgA1 production. In support of this hypothesis, it has been reported that the palatine tonsils of patients with IgAN have abnormal expression of enzymes involved in the glycosylation of IgA1 and that tonsil B cells themselves produce Gd-IgA1 [56,57,58,59]. The mechanism by which Gd-IgA1 is produced in the tonsils is shown in Figure 2. Furthermore, considering the increased proportion of mucinous IgA-positive plasma cells in the bone marrow in IgAN, it is possible that NALT-derived nephropathogenic B cells are stored in the bone marrow and systemic lymphoid tissues [60,61,62].

Recently, TNF superfamily member 13 (TNFSF13) has attracted attention as being part of the molecular mechanism involved in NALT activation [63]. TNFSF13 is one of the inducers of B-cell differentiation, is involved in T-cell-independent IgA production and IgA class switching, and is also called a proliferation-inducing ligand (APRIL) [64]. There are reports that APRIL levels in the blood are elevated in patients with IgAN [65]. In addition, the expression of APRIL and its receptors was increased in the palatine tonsils of patients with IgAN compared to patients with chronic tonsillitis, and APRIL positivity in GC correlated well with the severity of IgAN and response to tonsillectomy. APRIL positivity also correlated significantly with the rate of serum Gd-IgA1 reduction after tonsillectomy [66]. Furthermore, the involvement of TLR9 and CpG, a motif shared by bacteria with TLR9 ligands, in the enhancement of APRIL expression has been reported.

In recent years, tonsillectomy has been used as a treatment for IgAN, especially in Japan. A meta-analysis also supported the efficacy of tonsillectomy on IgAN [10], although several European studies did not report positive results of tonsillectomy on IgAN [67,68]. Because of epidemiological differences between Asia and Europe, such as the frequency ratio of dental disease and intestinal diseases, the degree of involvement of GALT and NALT in the pathogenesis of IgAN might differ among populations. Tonsillectomy and steroid therapy suppress activated NALT. Oral bacteria such as *S. mutans* related to dental caries and periodontal bacteria are suspected to stimulate NALT activation; nevertheless, the details are not known, but tonsillectomy can eliminate chronic bacterial infection in the tonsils.

## 5. GALT and IgA Nephropathy

In patients with inflammatory bowel diseases, such as ulcerative colitis and Crohn’s disease, an increase in mucosal-type IgA in the blood has been reported [69]. In these diseases, excessive IgA production due to intestinal inflammation and impaired pIgR expression results in increased mucosal-type multimeric IgA that is not transported to the mucosa and enters the circulating bloodstream. This multimeric IgA has a high affinity for the kidney and therefore deposits in the glomeruli, causing IgAN [69]. On the other hand, Kano et al. reported that there were no obvious differences in intestinal inflammation or IgA-positive regions between IgAN-onset ddY mice and quiescent ddY mice and that IgAN-prone ddY mice could develop IgAN without any intestinal changes [70]. In a mass spectrometry study, the number of galactose/hinge-part residues in serum IgA was significantly decreased in patients with IgAN compared to patients with Crohn’s disease and ulcerative colitis, suggesting that the glycosylation pattern of GALT-derived IgA is different from that of circulating nephropathogenic IgA [71]. In addition, the homing of B cells to mucosal and peripheral tissues is essentially controlled by a specific combination of chemokine receptors and adhesion molecules [52]. Differentiation of IgA-positive plasma cells in the lymphoid tissues of the small intestine induces upregulation of CC chemokine receptor 9 and α4β7 and homing back to the small intestine. In the small intestine, CC chemokine ligand 25 and mucosal adhesin cell adhesion molecule 1 are expressed [52]. Furthermore, the homing to colonic plasma cells is fine-tuned by the interaction of CC chemokine receptor 10 with CC chemokine ligand 28 [49]. Thus, it is generally believed that plasma cells generated from GALT remain localized in the intestine and cannot be transferred to the systemic immune system because of the combination of chemokine receptors and adhesion molecules. However, NALT induces α4β7, which interacts with vascular cell adhesion molecule 1 and induces the expression of L-selectin (CD62L), a systemic homing molecule, and CC chemokine receptor 7 [53].

In the 1970s and 1980s, there were reports that the intolerance of gluten, which is a type of protein produced from the endosperm of grains such as wheat and rye, was associated with the development of IgAN, and the relationship between celiac disease and IgAN has been discussed [72,73]. Coppo et al. reported that specific antibodies to gluten were elevated in patients with IgA nephropathy and that IgA nephropathy could be induced experimentally using gliadin, which is present in grains such as wheat and one of the glycoproteins that form gluten [74,75,76,77,78]. Recently, Papista et al. similarly reported gliadin-induced experimental IgA nephropathy [79]. Furthermore, in a mouse model expressing human IgA1 and CD89, early administration of a gluten-free diet improved the pathological features of the intestinal tract and prevented the development of IgA nephropathy [79]. In addition, the observation that a gluten-free diet reduces serum levels of IgA, including circulating immune complex, and IgA reactive to dietary antigens, suggests that it may correct immunological abnormalities in certain IgA nephropathy patients [80]. Other food antigens, such as gluten, might have pathogenicity of IgAN [76]. However, recent reports from the United States have shown negative results regarding the association between celiac disease and IgAN and that there are large regional differences in the development of IgAN due to celiac disease [81].

Some interesting data suggest that gut bacteria themselves may be able to regulate IgA class switch, mucosal IgA production, and O-galactosylation of IgA1 in GALT through activation of lymphoid TLRs [82,83]. Furthermore, there are reports indicating that the composition of the gut microbiota may be different in patients with IgA nephropathy [84,85]. In a large meta-analysis of genome-wide association studies, most of the risk alleles identified for IgA nephropathy were directly associated with either risk of inflammatory bowel disease, maintenance of the intestinal epithelial barrier, or response to mucosal pathogens. The geographic distribution of risk alleles strongly suggests multilocus acclimation, and genetic risk is strongly correlated with local enteric pathogen variation, suggesting that host–enteric pathogen interactions may be involved in shaping the genetic landscape of IgA nephropathy [86]. Moreover, enteric corticosteroid; budesonide ameliorated proteinuria in IgAN patients [87,88]. Based on these findings, gut–renal connection in IgAN had been proposed [89,90,91,92].

In terms of the relationship between oral bacterial infections and IgAN, living oral bacteria and components of oral bacteria should reach to the gastrointestinal tract; this may result in the cause of IgAN through GALT.

## 6. IgA Nephropathy and *Cnm*-(+) *S. mutans*: Clinical Evidence

*Streptococcus mutans* is known to be a major pathogen of dental caries [93]. Approximately 10–15% of *S. mutans* strains isolated from the oral cavity are shown to possess collagen-binding properties due to the presence of 120-kDa collagen-binding protein (*cnm*) encoded by the *cnm* gene [94,95]. *Cnm*-(+) *S. mutans* strains have the ability to bind to the extracellular matrix [96], which has been demonstrated to be associated with several diseases, such as infective endocarditis [97,98], aggravated cerebral hemorrhaging [11,15,16], inflammatory bowel disease [12] and nonalcoholic steatohepatitis [13,14]. In addition, recent studies have revealed that *cnm*-(+) *S. mutans* strains are also associated with the virulence of IgAN [94,95,99,100,101,102].

One of the clinical studies demonstrated that the positive rate of *cnm*-(+) *S. mutans* in saliva specimens was significantly greater in the IgAN group than in the control group (32.1 vs. 14.0%), although the positive rates of isolation of *S. mutans* between the IgAN and control groups were almost the same [94]. Another study showed that the number of dental caries and the urinary protein levels in the *cnm*-(+) *S. mutans* group were significantly higher than those in the *cnm*-negative *S. mutans* group. The study also demonstrated that the urinary protein levels in the high dental caries experienced group were significantly higher than those in the low group, suggesting that *cnm* positivity and dental caries status are associated with urinary protein levels in IgAN patients [95]. On the other hand, *Campylobacter rectus*, a periodontitis-related bacterial species in the oral cavity, has also been shown to be significantly associated with proteinuria in IgAN patients [99], which suggests the possibility that multiple oral bacterial species could be associated with the pathogenesis of IgAN [99].

The precise mechanism by which the presence of *cnm*-(+) *S. mutans* in the oral cavity induces exacerbation of IgAN remains to be elucidated. There is a report describing that the *Cnm* protein in the tonsils may be associated with severe IgAN [100]. This report showed that: (1.) the *Cnm* protein-positive area/total tonsillar area ratio was significantly higher in patients with IgAN than in the control (chronic tonsillitis) group; (2.) *Cnm* protein was observed in the tonsils but not the kidneys of IgAN patients; and (3.) *Cnm* protein expression in the tonsils was associated with the urinary protein levels of IgAN patients. These results suggest that IgAN may be exacerbated via the immune response of the tonsils by unknown effects caused by the *Cnm* protein, which will be continuously supplied from saliva to the tonsils [100].

## 7. IgA Nephropathy and *Cnm*-(+) *S. mutans*: Animal Models

Several reports have indicated that intravenous administration of *S. mutans* into rabbits led to severe nephritis involving glomeruli, tubules, and interstitium [103,104,105], reporting their observations of glomeruli with documented diffuse endocapillary proliferative glomerulonephritis often accompanied (65%) by epithelial crescents and fibrin deposits with sclerosis evident in the glomeruli of some rabbits, which is characteristic of poststreptococcal glomerulonephritis and is distinct from that of typical IgAN, which is mesangial glomerulonephritis. At that time, the *Cnm* protein had not yet been discovered, and the tested *S. mutans* strain was *Cnm*-negative. However, this model might be helpful in understanding the mechanism of nephritis via infection of *S. mutans*.

**Figure 3:**
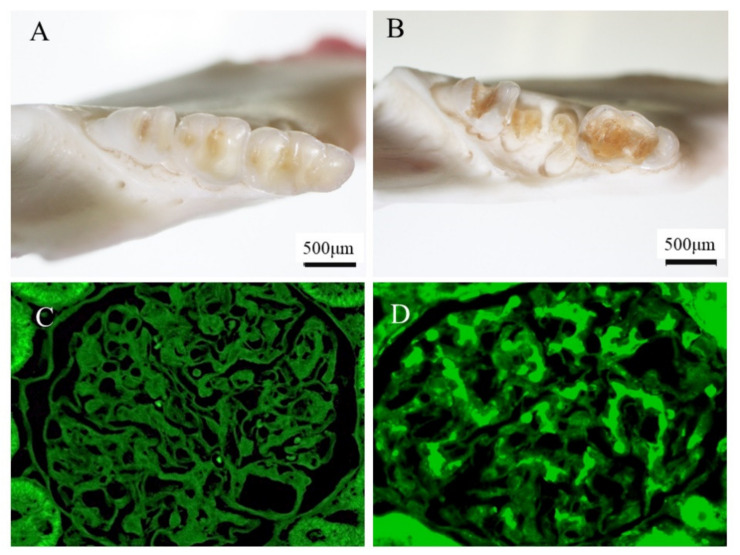
**IgA nephropathy model rat induced by *cnm*(+)-*S. mutans*.** In a mouse model, dental caries induced by *cnm*(+)-*S. mutans* could cause mesangial IgA deposition, which is an important feature of IgAN. Details of the methods and the characteristics of the model rats are described in a previous reference [100]. These figures were originally prepared for this review. (**A**) Control mouse teeth. (**B**) Dental caries in mice treated with *cnm*(+)-*S. mutans*. (**C**) There was no IgA deposition in the glomeruli of control mice. (**D**) IgA deposition in the glomeruli of mice treated with *cnm*(+)-*S. mutans*.

Recently, some experimental IgAN-like nephritis models via infection of *cnm*-(+) *S. mutans* have been reported [101,102]. One of the models is a rat model administered *cnm*-(+) *S. mutans* via the jugular vein, which demonstrated transient induction of IgAN-like lesions at 30 and 45 days and recovered at 60 days [101]. This model demonstrated transient proteinuria, mesangial cell and matrix proliferation, mesangial IgA and C3 deposition and partial C3 subendothelial deposition by fluorescence immunostaining, as well as mesangial deposition and a hump in electron microscopy analyses, all of which are characteristics of both IgAN and IgA-dominant infection related to glomerulonephritis [101]. Another model is rats with severe dental caries induced by inoculation with a *cnm*-(+) *S. mutans* strain in the oral cavities of 2-week-old specific-pathogen-free rats that were fed a high-sucrose diet for 32 weeks, in which IgAN-like glomerulonephritis was identified (see Figure 3) [102]. This model demonstrated persistent hematuria, mild mesangial cell and matrix proliferation, and mesangial IgA and C3 deposition by fluorescence immunostaining (see Figure 3), as well as mesangial deposition in electron microscopy analyses.

Although further studies are required to elucidate the detailed mechanisms, it is expected that the mechanism of IgAN will be clarified by the *cnm*-(+) *S. mutans* infection model in the near future.

## 8. IgA and Periodontal Bacteria

**Figure 4:**
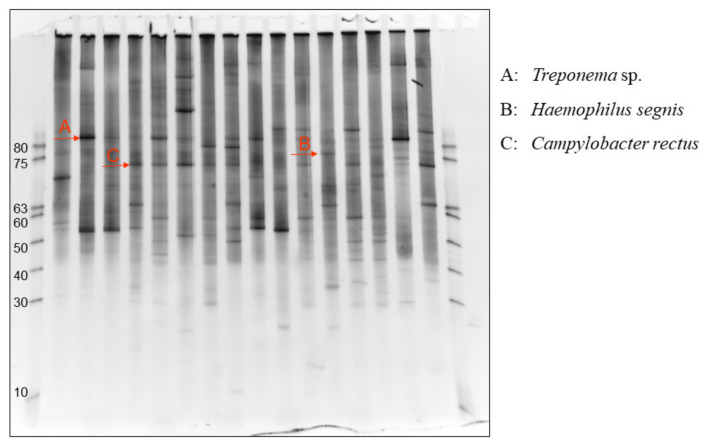
**Results of DGGE analysis using mRNA from the tonsils of IgAN patients.** Denaturing gradient gel electrophoresis (DGGE) can provide each band specific to each bacterium using universal PCR primers and bacterial ribosomal RNA. The intensity of the band usually semi-quantitatively reflects the amount of the bacteria. Therefore, in DGGE methods, the position of the bands indicates the type of bacteria, and the intensity of the bands indicates the amount of the bacteria. Red arrows indicate specific bands to A: *Treponema species*, B: *Haemophilus segnis*, and C: *Campylobacter rectus*. This figure was originally prepared for this review.

As the stimulator of IgA production in the tonsils or oral area, bacteria are the best candidate because acute bacterial infection sometimes causes macrohematuria. There are several problematic points in typical culture methods. First, culture methods cannot detect anaerobic bacteria. Second, each bacterium usually requires a specific culture medium. Third, the amounts of bacteria cannot be evaluated by the method. Denaturing gradient gel electrophoresis (DGGE) can overcome these disadvantages [106]. Using universal PCR primers in bacterial ribosomal RNA, each PCR product from each bacterium yields specific bands after denaturing gradient gel electrophoresis (see Figure 4) [107]. The intensity of the band usually semi-quantitatively reflects the amount of the bacteria. Therefore, in DGGE methods, the position of the bands indicates the kinds of bacteria, and the intensity of the bands indicates the number of bacteria (see Figure 4). Using this information, the specific bacteria in the tonsils of IgAN patients can be elucidated and compared with those in tonsilitis patients. After the specific bands in IgAN patients were identified, sequencing of the bands can reveal bacterial species because each bacterium usually has one specific ribosomal RNA. Using DGGE methods, *Treponema denticola*, *Hemophilus segnis,* and *Campylobacter rectus* were identified as specific bacteria in the tonsils of IgAN patients. Moreover, among these bacteria, *T. denticola* and *C. rectus* had a strong association with remission of proteinuria and hematuria induced by combination therapy with tonsillectomy and steroid pulse [107]. *T. denticola* and *C. rectus* are well known to be pathogenic periodontal bacteria; therefore, periodontal bacteria have become candidates as pathogenic stimulators of IgAN [107]. The relationship between IgAN patients and *C. rectus* was confirmed in another cohort, although the detection rate of *T. denticola* was too low to evaluate the relationship [99]. In this report, double infections of *C. rectus* and *cnm*(+)-*S. mutans* were associated with the severity of proteinuria, which also supported the pathogenicity of IgAN induced by *C. rectus*.

**Figure 5:**
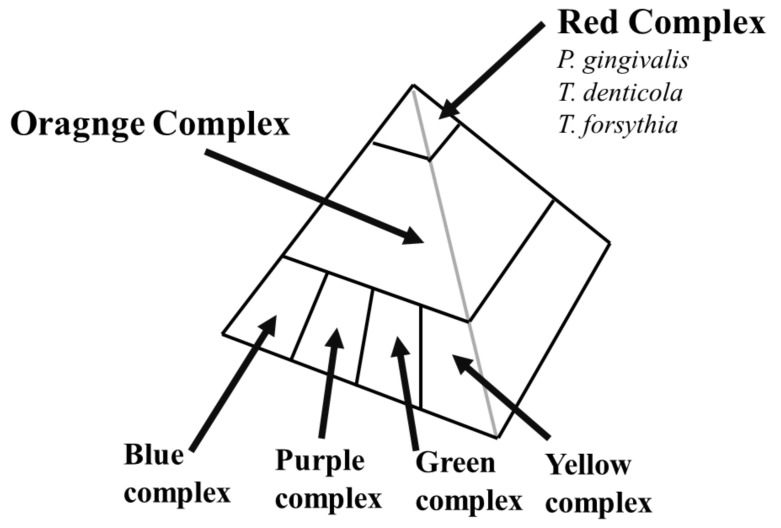
**Pyramid structure of periodontal bacteria.** First, periodontal bacteria belong to the blue, purple, green, and yellow complexes colonizing pockets around teeth. These periodontal bacteria have a relatively low level of periodontal disease pathogenicity. Second, the orange complex of periodontal bacteria colonizes the pockets. Finally, red complex periodontal bacteria, such as *P. gigivalis, T. denticola,* and *T. forsythesis*, can colonize the pockets, which have strong periodontal disease pathogenicity.

The crypts in the tonsils are an anaerobic environment, as are the pockets around teeth. Therefore, periodontal bacteria can move to the tonsils from the pockets around the teeth. *T. denticola* belongs to the red complex of periodontal bacteria, which has the strongest periodontal disease pathogenesis (see Figure 5). *C. rectus* belongs to the orange complex of periodontal bacteria, which has the second strongest pathogenesis (see Figure 5). After periodontal bacteria are identified as candidates in the pathogenesis of IgAN, pathogenic periodontal bacteria should be investigated systemically. Familial IgAN patients have been reported. Basically, the pathogenesis of familial IgA is usually accounted for by genetic factors. However, periodontal bacteria can also account for the pathogenesis of familial IgA because periodontal bacteria can often transfer from mother to child.

Another group also tried to comprehensively analyze specific bacteria in the tonsils from IgAN patients using a high-throughput multiplexed sequencing approach [108]. Genomic DNA from the tonsillar crypts of each patient was extracted, and the V4 regions of the 16S ribosomal RNA gene were amplified and analyzed to determine the bacteria. However, it was difficult to determine the species of bacteria beyond the bacterial genus; therefore, there were no significant differences in the microbiome composition among the groups of IgAN patients according to clinicopathological parameters [108]. Another study recently focused on the relationship between oral microbiomes and systemic diseases [109]. In terms of relationship between oral microbiomes and IgAN, there have been several positive reports [47,110]. However, on this point, specific bacteria rather than dental caries bacteria and periodontal bacteria had not been identified.

## 9. IgA and Periodontal Bacteria: Animal Models

The periodontal bacteria represent many categories of periodontal disease pathogenicity (Figure 5). Among these bacteria, the red complex of bacteria has the strongest periodontal disease pathogenicity. Recently, *P. gingivalis*, from the red complex of periodontal bacteria, was reported to be significantly more highly detected in IgAN patients than in habitual tonsilitis patients [111]. *P. gingivalis* colocalizes with *C. rectus*, an orange complex periodontal bacterium, and *C. rectus* has been reported to be highly detected in the tonsils of IgAN patients [112]; moreover, *C. rectus* has a relationship with the clinical remission rate after tonsillectomy [107], indicating that *C. rectus* infection might be accompanied by *P. gingivalis* infection, which has strong IgAN pathogenicity.

IgA model mice induced by *P. gingivalis* have recently been reported [111]. *P. gingivalis* might have difficulty establishing chronic infection because it is anaerobic. Therefore, nasal administration of *P. gingivalis* to mice was continued for eight weeks. Nasal administration of *P. gingivalis* in mice caused mesangial proliferation (*p* < 0.05 at days 28 and 42 and *p* < 0.01 at days 14 and 56) and IgA deposition (*p* < 0.001 at days 42 and 56 after administration). Scanning electron microscopy revealed that high-density electron-dense deposition was widely distributed in the mesangial region in the mouse kidneys treated with *P. gingivalis.* These histological features are specific to IgAN. These findings suggest that *P. gingivalis* is involved in the pathogenesis of IgAN.

There were many unresolved questions regarding the IgA mouse model induced by *P. gingivalis*. Is it *P. gingivalis* bacterial infection itself or a component of *P. gingivalis* that is involved in the pathogenesis of IgAN? Double infections might have stronger pathogenicity than single infections because there were multiple infections of periodontal bacteria in the tonsils of IgAN patients. Moreover, the mechanism by which periodontal bacterial infection causes aberrantly glycosylated IgA1 remains unknown. In the future, detailed mechanisms should be revealed using these animal models.

## 10. IgA and Cigarette Smoking

**Figure 6:**
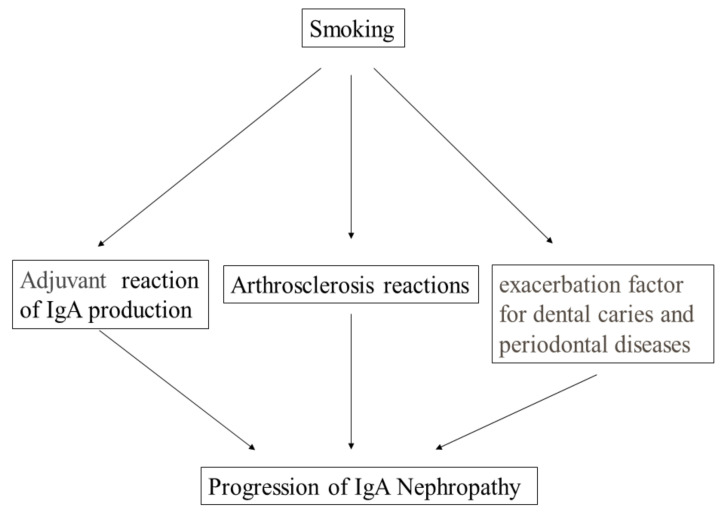
**Mechanism by which smoking is considered to accelerate the progression of IgAN.** Smoking has been reported to accelerate the progression of IgAN. Classically, this acceleration was explained by atherosclerotic reactions because many atherosclerotic factors can worsen CKD. As another potential mechanism, chemicals present in cigarettes can work as chemical adjuvants, which might increase IgA production in the oral mucosal area. As another considered mechanism, smoking can worsen both dental caries and periodontal diseases, resulting in an increase in the production of pathogenic IgA.

Smoking worsens the progression of IgAN [7]. Moreover, in IgAN patients with advanced chronic kidney disease (CKD) stages, the deleterious effect induced by smoking becomes exacerbated compared to that in IgAN patients with normal kidney function [113]. The harmful effect on the progression of CKD induced by smoking and the beneficial effect of cessation of smoking were similar to the effect on the incidence of cancer rather than the effect on the incidence of cardiovascular diseases [114]. The mechanism of these effects on the progression of IgAN has basically been explained by metabolic reactions caused by toxic substances in cigarette smoking (see Figure 6), similar to the mechanism of these effects on the incidence of cancers [114]. Another potential mechanism specific to IgAN is the role of smoking as a chemical adjuvant. Obviously, smoke reaches the tonsils and the epipharynx, which contain NALT. Adjuvant molecules enhance the production of antibodies, resulting in the exacerbation of IgAN (see Figure 6).

Another mechanism involves the relationship between smoking and oral bacteria. Many reports, including meta-analyses, have indicated that smoking worsens dental caries [115,116]. Similarly, many reports have supported that smoking worsens periodontal disease [117]. These findings indicated that smoking worsens oral pathogenetic bacteria. These bacteria can activate NALT and IgA production. Elevated NALT sometimes causes Gd-IgA production, resulting in the incidence of IgAN (see Figure 6).

Cession of smoking is encouraged for the general population because cessation of smoking inhibits the incidence of both cancer and cardiovascular events, resulting in reduced mortality. In CKD patients, cessation of smoking also improved renal prognosis, although the effect on renal survival took a longer period than the effect on cardiovascular events. Cession of smoking improves the recovery from dental caries and periodontal bacteria [117]. This effect should ameliorate the origin of IgAN, which can be expected to improve renal prognosis. For IgAN patients, cessation of smoking is highly recommended.

Smoking is also a detrimental factor for renal survival and mortality in renal transplantation patients [118]. Not surprisingly, smoking increases respiratory infections, which are sometimes fatal for transplantation patients. Moreover, smoking might cause the recurrence of IgAN in renal transplantation patients whose original kidney disease was IgAN through worsening of oral bacteria, such as dental caries and periodontal bacteria. Recently, tonsillectomy was reported to improve urinary findings in renal transplantation patients [119,120], which might indicate that the treatment eliminated periodontal bacteria from NALT. Cession of smoking is also highly recommended in renal transplantation patients to improve renal survival and mortality through the amelioration of oral bacteria.

## 11. Hypothesis of the Mechanism by Which Oral Infection Induces Aberrantly Glycosylated IgA1, Which May Induce IgA Nephropathy

**Figure 7:**
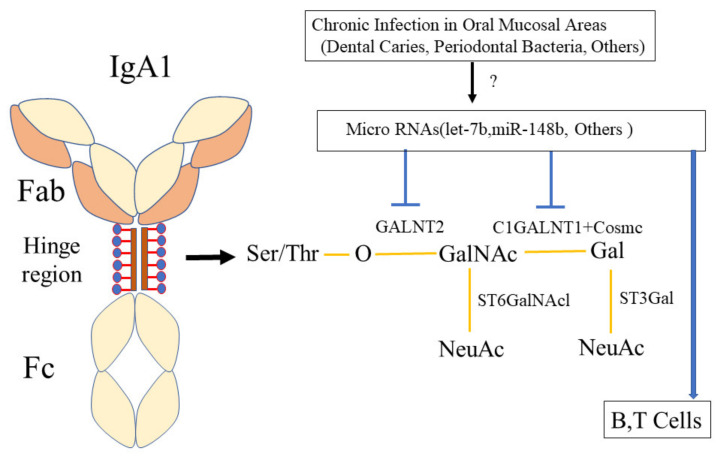
**Hypothesis of the mechanism by which oral bacterial infection causes aberrantly glycosylated IgA1.** The hinge region of IgA1 contains serine (Ser) or threonine (Thr) amino acids, which can be glycosylated. This glycosylation contributes to the aggregation of IgA1 because of the reactivity of IgA1 to jacalin, a lectin specific for O-linked glycan side chains containing GalNAc, N-acetyl-galactosamine (GalNAc) and galactose (Gal) linked by beta-1,3-glycosidic bonds. These O-glycan chains in the IgA1 hinge region are formed by sequential reactions that are catalyzed by some glycosyltransferases, such as GALNT2, C1GALT1, and COSMC. The expression levels of these enzymes were altered by microRNAs, such as let-7b and miR-148b, resulting in aberrantly glycosylated IgA1. The expression of these microRNAs might be altered by chronic infections in the oral mucosal areas, such as bacteria related to dental caries and periodontal bacteria. Ser: serine amino acid, Thr: threonine amino acid, GalNAc: N-acetyl-galactosamine, GALNT2: UDP-N-acetyl-alpha-D-galactosamine:polypeptide N-acetyl-galactosaminyl-transferase 2, C1GALT1: core 1 beta-1,3-galactosyltransferase, COSMC: core 1-β3 galactosyl-transferase specific molecular chaperone.

IgAN is defined by predominant IgA deposition in the mesangial areas, and the subclass of IgA in the mesangial area is IgA1 [2,5]. IgA1 has a hinge region, whose serine (Ser) or threonine (Thr) amino acids can be glycosylated (see Figure 7) [121,122]. This glycosylation contributes to the aggregation of IgA1 because of the reactivity of IgA1 to jacalin, a lectin specific for O-linked glycan side chains containing N-acetyl-galactosamine (GalNAc) and galactose (Gal) linked by beta-1,3-glycosidic bonds. These O-glycan chains in the IgA1 hinge region are formed by sequential reactions catalyzed by some glycosyltransferases, such as UDP-N-acetyl-alpha-D-galactosamine:polypeptide N-acetyl-galactosaminyl-transferase 2 (GALNT2), core 1 beta-1,3-galactosyltransferase (C1GALT1), and core 1-β3 galactosyl-transferase specific molecular chaperone (COSMC) (see Figure 7) [121,122]. In IgAN patients, aberrant glycosylation of the IgA1 hinge region has been reported. Moreover, decreased expression levels of these glycosylation enzymes have been reported. However, there were no differences in the genetic backgrounds, such as single-nucleotide polymorphisms (SNPs) in the genes coding these glycosylation enzymes [123].

MicroRNAs have been reported to alter the expression levels of these glycosylation enzymes, which are related to the pathogenicity of IgAN. The microRNA let-7b can inhibit GALN2 expression [124] (see Figure 7). Another microRNA, miR-148b, can inhibit C1GALT1 [125] (see Figure 7). Moreover, serum levels of miR-148b and let-7b were related to the response to treatment and long-term outcome in IgAN [126]. The microRNA miR-196b can affect COSMC [127]. These microRNAs can directly affect the glycosylation of IgA1. MicroRNA miR-320 promotes B-cell proliferation, resulting in the production of aberrantly glycosylated IgA1 [128]. The microRNA miR-374b also promotes cell proliferation and the production of aberrantly glycosylated IgA1 in B cells of IgAN subjects (see Figure 7) [129]. Another microRNA, miR-155, induced T-cell subgroup drifting in IgAN [130]. The microRNA miR-98-5p also affected the glycosylation of IgA1 through cytokines [131], while miR-630 regulated underglycosylated IgA1 in tonsils by targeting TLR4 in IgAN [131]. These microRNAs can affect T or B cells, resulting in altered glycosylation of IgA1. There is still the possibility that many other microRNAs might be related to IgA1 glycation abnormalities. After tonsillectomy and steroid pulse therapy, the IgA1 abnormality reverted to a normal distribution [51], which also supports the variability of IgA1 abnormalities induced by microRNAs. However, the therapy could not completely correct the distribution of IgA1 glycations, probably because other portions rather than tonsils, such as the epipharynx, can also produce IgA1 in the upper respiratory tract. Indeed, hematuria and proteinuria after tonsillectomy can be ameliorated by disinfection of the epipharynx [132].

In terms of the pathogenesis of IgAN, it is important to reveal direct evidence between chronic oral bacterial infection and microRNAs, resulting in aberrant glycosylation of IgA1 (see Figure 7). Currently, the precise mechanism of the regulation of microRNAs is unknown. Therefore, there are no reports that have proved the relationship between oral bacterial infection and microRNA expression. IgA1 in the oral area is produced to eliminate infectious bacteria. Therefore, it is reasonable that oral bacteria, such as *cnm*(+)-*S. mutans* and periodontal bacteria, could alter the microRNAs to properly produce IgA1, sometimes resulting in the production of aberrant glycosylation of IgA1. Aberrantly glycosylated IgA1 induced anti-aberrantly glycosylated IgA-IgG [133]. Aberrantly glycosylated IgA1 aggregates with itself, and the immune complex of aberrantly glycosylated IgA1 and anti-aberrantly glycosylated IgA1-IgG deposits in the mesangial area in the glomeruli, resulting in the incidence of IgAN.

## 12. Gene Influence on The Relationship between IgA and Infection

Familial IgAN patients are sometimes observed in daily clinical practice. Additionally, ddY mice are known IgA model mice that spontaneously exhibit IgAN-like clinical symptoms. These findings indicate that there are some genetic factors contributing to the incidence of IgAN. Indeed, the locus associated with IgAN was reported in 2000 [134] using classical pedigree analysis, and this locus was confirmed by a mouse ddY model [65]. The candidate gene approach also revealed many genes contributing to the incidence of IgAN [135,136]. A genome-wide association study (GWAS) revealed new risk loci for IgAN that might be involved in immunity against intestinal pathogens [86]. This group proposed that the combination of human leukocyte antigen (HLA) and IgA1-O-glycation, innate immunity, the complement system, and environmental factors caused IgAN [137]. As environmental factors, intestinal pathogens from bacteria have been proposed in these reports [86,137]. Likewise, oral bacteria could be environmental factor candidates because pathogens from oral bacteria can influence immune cells, similar to pathogens from intestinal bacteria. The response to bacterial infection in NALT might be altered by genetic factors, which might already be revealed.

The distribution of periodontal bacteria in children often corresponds to that in mothers because periodontal bacteria can transfer from mother to children through mouth-to-mouth feeding [138]. The frequency and intensity of oral care are usually the same in a family, which could cause the same level of dental caries or periodontal diseases. These findings indicated that oral bacteria, such as periodontal bacteria and bacteria related to dental caries, could transfer from a mother to her children, similar to genetic factors. This oral bacterial factor might disturb genetic analyses, but recent interpretations based on GWASs concluded that IgAN might be caused by both genetic factors and environmental factors, such as bacteria in the intestine [137], or more likely in the oral areas.

## 13. Conclusions Regarding Oral Bacteria and IgA Nephropathy

The relationship between oral bacterial infection and IgAN has been suspected. Recent studies have indicated that chronic oral bacterial infections, such as *cnm*(+)-*S. mutans* and periodontal bacteria, might be involved in the pathogenesis of IgAN. Establishment of rat or mouse models of IgAN induced by *cnm*(+)-*S. mutans* or periodontal bacteria might reveal the precise mechanism of the pathogenicity induced by these bacteria in the future. Regardless, although the mechanism by which chronic oral bacterial infections cause IgAN remains unknown, oral care should be recommended for IgAN patients.

## Data Availability

The data presented in this study are available upon request from the corresponding author. The data are not publicly available, due to ethical and privacy limitations.
